# Epidemiology of Paediatric Shoulder Dislocation: A Nationwide Study in Italy from 2001 to 2014

**DOI:** 10.3390/ijerph17082834

**Published:** 2020-04-20

**Authors:** Umile Giuseppe Longo, Giuseppe Salvatore, Joel Locher, Laura Ruzzini, Vincenzo Candela, Alessandra Berton, Giovanna Stelitano, Emiliano Schena, Vincenzo Denaro

**Affiliations:** 1 Department of Orthopaedic and Trauma Surgery, Campus Bio-Medico University, Via Alvaro del Portillo, 200, 00128 Rome, Italy; g.salvatore@unicampus.it (G.S.); h.locher.joel@gmail.com (J.L.); v.candela@unicampus.it (V.C.); a.berton@unicampus.it (A.B.); g.stelitano@unicampus.it (G.S.); denaro@unicampus.it (V.D.); 2 Department of Orthopedics, Children’s Hospital Bambino Gesù, Via Torre di Palidoro, Palidoro, 00165 Rome, Italy; laura.ruzzini@opbg.net; 3 Unit of Measurements and Biomedical Instrumentation, Università Campus Bio-Medico di Roma, Via Alvaro del Portillo, 21, 00128 Rome, Italy; e.schena@unicampus.it

**Keywords:** epidemiology, shoulder, dislocation, surgery, reduction, instability, young, child, children

## Abstract

Limited knowledge is accessible concerning the tendencies of hospitalization for skeletally immature patients with episodes of shoulder dislocation. Our research aim was to evaluate annual hospitalizations for shoulder dislocation in paediatric patients in Italy from 2001 to 2014, on the basis of the official data source as hospitalization reports. The second purpose was to investigate geographical diversification in hospitalization for shoulder dislocation in regions of Italy. The last aim was to make statistical predictions of the number of shoulder dislocation hospitalization volumes and rates in skeletally immature patients based on data from 2001 to 2014. An examination of the National Hospital Discharge records (SDO) kept at the Italian Ministry of Health regarding the 14 years of our study (2001 through 2014) was conducted. These data are anonymous and include patient’s age, gender, domicile, region and time of hospitalization, and the kind of reimbursement (public or private). In the 14-year study time, 344 hospitalizations for shoulder dislocation of patients aged 0–14 years took place in Italy. The male/female hospitalization ratio varied from a maximum of 3.0 (2001) to a minimum of 1.1 (2013), with a mean average ratio in the 2001–2014 timespan of 2.0. Almost half of the hospitalizations (49.1%) were performed in the South. The mean incidence of hospitalizations in Italy for shoulder dislocation in patients with less than 14 years was 0.3 for every 100,000 inhabitants in the same class of age. The most common treatment was a closed reduction (60.8%), followed by open stabilization (16.6%) and arthroscopic procedures (13.7%). The present registry study shows a low incidence of hospitalization for shoulder dislocation in young patients. The most common treatment for a shoulder dislocation in paediatric patients is a closed shoulder reduction. Regions from the south and the centre of Italy are marked by an inferior number of operations and a higher number of hospitalization for closed shoulder reduction.

## 1. Introduction

Shoulder dislocations are common, with a rate of around 23.9 per 100,000 pople a year [1,2,3,4]. In young subjects with an immature skeleton, physes remain open and shoulder injury may lead to physeal or metaphyseal fractures rather than dislocation [5,6,7,8]. Nevertheless, 40% of shoulder dislocations happen in subjects younger than 22 [1,9,10,11]. Subjects under the age of 20 are at most significant risk of developing a shoulder dislocation, while the same pathology is very uncommon in infants younger than ten years [1,2,11,12,13,14]. These diseases are particularly diffuse among young athletes, with rate as high as 7% among hockey players [15,16,17,18,19]. Young patients also have an elevated risk of developing recurrent glenohumeral instability compared to older patients [4,9,20,21].

The decision between conservative versus operative treatment for shoulder dislocation and the proper timing for surgery in youthful subjects is still discussed [9,22,23,24,25,26]. A conservative method has generally been defined the gold standard for a primary shoulder dislocation in young patients, but recent evidence suggests that early surgical procedures result in the reduction in subsequent episodes of glenohumeral instability [20,25,26,27,28,29,30,]. In current literature, there are a few studies examining shoulder instability only in paediatric, and especially in skeletally immature, patients [11,31,32].

Despite several publications addressing shoulder dislocation aetiology [13,33,34,35,36], indications for operative treatment [14,37,38], and results in specific groups of subjects [10,11,13,18,36,37,38,39], limited knowledge is accessible relating to tendencies of hospitalization for skeletally immature patients with episodes of glenohumeral instability. We are not aware of any available database or registry on this population.

An understanding of the trends of hospitalization for shoulder instability in skeletally immature patients could be essential to adequately guide the future development of this condition and any associated health assistance plan.

The Italian National Health Service was established in 1978. Hospitalization and surgical procedures are completely free of charge for all citizens, regardless of income. The National Health Service is administered on a regional basis [40]. Important variations in the quality and outcomes of care by region have been reported, as well as patient movement from southern to northern regions, probably driven by a search for better healthcare quality [41,42,43].

This study assesses to estimate the yearly number of hospitalizations for shoulder dislocation in skeletally immature patients in Italy from 2001 to 2014, on the basis of official data resources and hospitalization reports. Our second purpose was to investigate geographical diversification in treatment and hospitalization for shoulder dislocation in regions of Italy. The last goal was to make statistical predictions of the amount of shoulder dislocation hospitalization volumes and rates in skeletally immature patients based on data from 2001 to 2014.

## 2. Materials and Methods

An examination of the National Hospital Discharge reports (SDO) reported at the Italian Ministry of Health concerning the 14 years of the present research (2001 through 2014) was conducted. The SDO collects data on every hospitalization happening in the Italian public and private sectors. This information is anonymous and incorporate patient’s gender, age, domicile, region, and where the hospitalization was performed. National and regional population information was collected from the National Institute for Statistics (ISTAT) annually. Shoulder dislocation was determined by the subsequent International Classification of Diseases, Ninth Revision, Clinical Modification (ICD-9-CM) primary diagnosis code: 831.00 (Closed dislocation of shoulder, unspecified), 718.31 (Recurrent shoulder dislocation), 831.09 (Closed dislocation of shoulder, other). An analysis of shoulder dislocation in young patients was performed. We defined “young”—in agreement with the Italian National Institute for Statistics (ISTAT)—as patients aged between 0–14 years. The considered treatment codes were: 81.82: Reparation of recurrent dislocation of the shoulder; 81.83: Other shoulder repair; 79.71: Closed Shoulder reduction; 79.81: Open Shoulder reduction; 80.21: Shoulder Arthroscopy.

### 2.1. Population

We divided patients on the basis of their domicile to identify “regional populations”. Furthermore, we distinguished for any country the origin of subjects who underwent an operation. Methods conducted on subjects living in the same area of hospitalization were designated as “regional surgeries”. Methods conducted on subjects not living in the same country of hospitalization were designated as “extra-regional surgeries”.The national and regional incidence of hospitalization was obtained by dividing the number of hospitalizations of patients aged 0–14 years and the national/regional population in the same age-class respectively.

### 2.2. Regions of Italy

**Figure 1:**
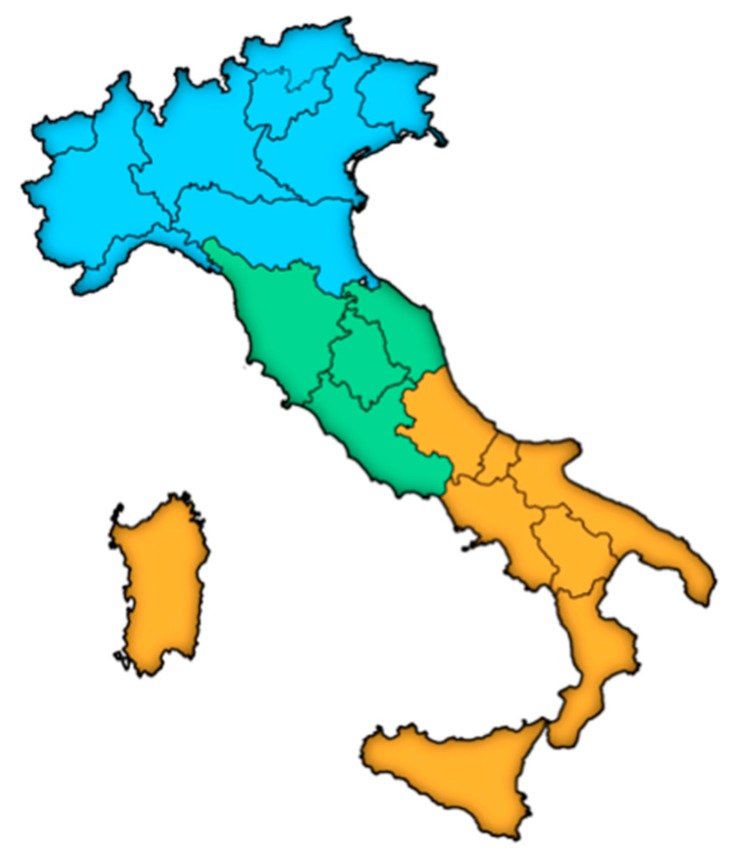
Italian Geographical macroregions are the North (blue), the Centre (green) and the South (brown).

The North of Italy comprises the countries of the North-West (Liguria, Lombardy, Piedmont, Aosta Valley) and the regions of the North-East (Emilia-Romagna, Friuli-Venezia Giulia, Autonomous Province of Trento, Autonomous Province of Bolzano, Veneto). The center incorporates the regions of Lazio, Marche, Tuscany, and Umbria. The South consists of the areas of Southern Italy (Abruzzo, Basilicata, Calabria, Campania, Molise, Apulia) and the islands (Sardinia and Sicily). (Figure 1).

### 2.3. Surgical Hospitalization Rates

We divided the hospitalizations into 2 main categories: “non-surgical hospitalization” and “surgical hospitalization”. Non-surgical hospitalizations were those in which the procedures performed did not require any surgery, thus they could have been performed as an outpatient procedure. Surgical hospitalizations were those that required surgery. For the aim of this study, the code 79.71 (Closed Shoulder reduction) was considered as non-surgical treatment. The codes 81.82: Reparation of recurrent dislocation of the shoulder; 81.83: Other shoulder repair; 79.81: Open Shoulder reduction; and 80.21: Shoulder Arthroscopy were considered as surgical treatment.

### 2.4. Statistics

Descriptive statistical analyses were applied to determine the yearly amount of hospitalizations for gleno-humeral instability in the young Italian population (0–14 years old). Incidence was estimated utilizing the annual juvenile population proportion of the same age class (0–14 years old) taken from ISTAT, a statutory electronic national population register. Projection was conducted utilizing the “Forecast” function in Excel (Microsoft) software.

## 3. Results

### 3.1. Demographics

In the 14-year study time, 344 hospitalizations for shoulder dislocation of patients aged 0–14 years were performed in Italy.

The male/female hospitalization ratio varied from a maximum of 3.0 (2001) to a minimum of 1.1 (2013), with a mean average ratio in the 2001–2014 timespan of 2.0. (Table 1).

The number of procedures per 100,000 inhabitants were showed in Table 2.

The mean national incidence in the 14-year study period resulted in 0.3 hospitalizations for every 100,000 inhabitants of the same age class.

From 2001 to 2014, the incidence of hospitalizations decreased from 0.39 per 100,000 inhabitants of the same age in 2001 to 0.25 per 100,000 inhabitants of the same age in 2014 (Table 3).

Almost half of the overall hospitalizations (49.1%) were performed in Southern Italy (Table 4), with the majority being performed in the period 2001–2006.

Inhabitants of Autonomous Province of Bolzano, Molise, Sicily, and Apulia resulted to have the greater rate of hospitalization with respect to the other regions of Italy (Table 5).

**Figure 2:**
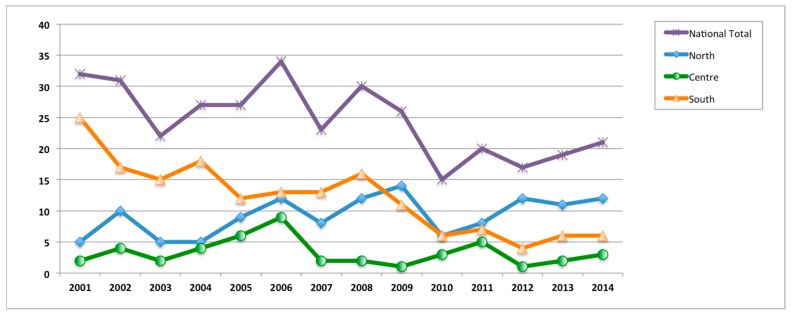
Hospitalization differences among Italian macro-areas (n° hospitalizations/year).

Schematic representation of hospitalization differences among Italian macro-areas is represented in Figure 2.

**Figure 3:**
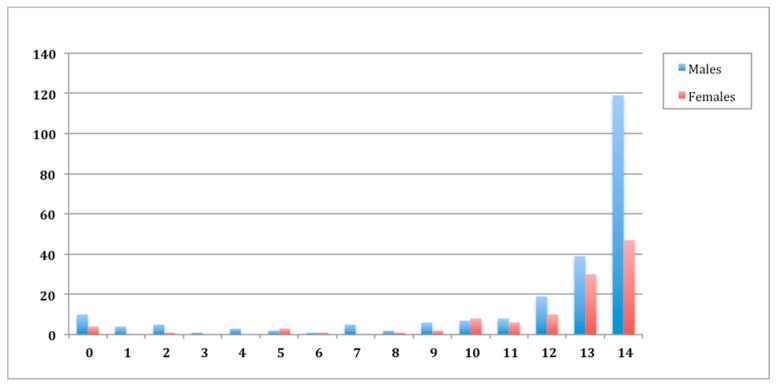
Hospitalizations in 2001–2014 timespan, stratified by patient age when the hospitalization was performed.

The number of hospitalizations to treat shoulder instability in Italy in the research time by age groups is reported in Figure 3. Across the research time, the greatest rate of hospitalizations was observed at the age of 14.

More than 75 % (*n* = 264) of hospitalizations to treat shoulder instability were performed in males from 12 to 14 years of age.

Of 344 hospitalizations for shoulder dislocation conducted in Italy in the research time, domicile information were not available for seven patients. Therefore, there were finally 337 hospitalizations for which patients’ domicile data were detected. (Table 2).

### 3.2. Region of Hospitalization

Concerning regional dislocation of hospitalizations, the majority of subjects were hospitalized in their domicile. Ten out of 21 regions of Italy resulted to have a regional hospitalization percentage greater than 85%. Aosta Valley resulted the only Region that did not perform any regional hospitalization in the analysed period. Data on interregional migration, regional, and extra-regional hospitalizations are summarised in Table 2.

### 3.3. Admission Diagnosis and Procedure Performed

**Figure 4:**
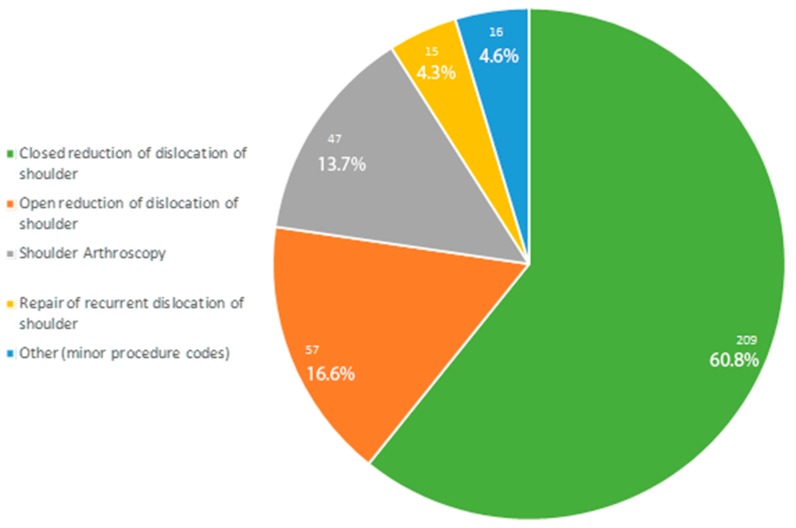
Pie chart of treatment codes.

**Figure 5:**
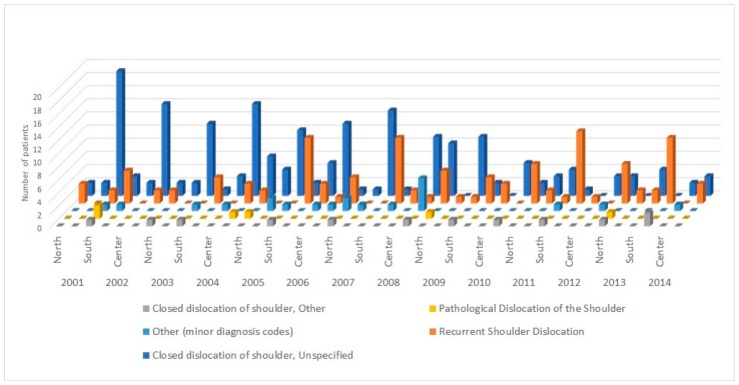
Data on admission diagnosis performed by macroregion of Italy and by year.

**Figure 6:**
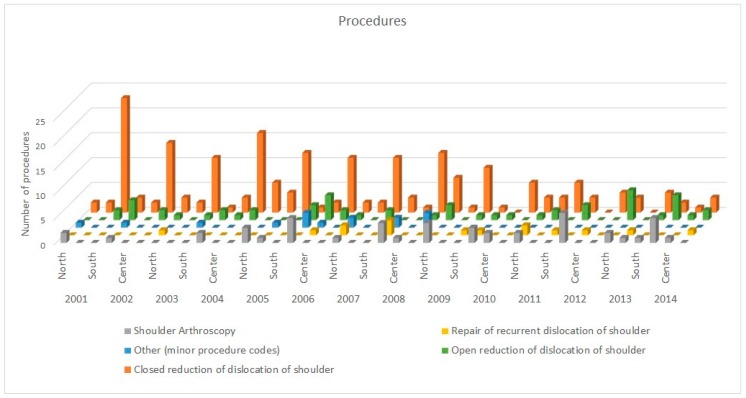
Data on surgical procedures performed by macroregion and by year.

Data obtained from the registry showed that the major admission diagnosis code was by far the 83.100 (“Closed dislocation of shoulder, Unspecified”), representing alone more than 55% of admission diagnoses. Regarding the procedures performed during the hospitalization, more than 95% of patients underwent one of the following procedures: “Closed reduction of dislocation of shoulder” (*n* = 209, 60.8%), “Open reduction of dislocation of shoulder” (*n* = 57, 16.6%), “Shoulder Arthroscopy” (*n* = 47, 13.7%), “Repair of recurrent dislocation of shoulder” (*n* = 15, 4.4%) (Figure 4). Data on the regional distribution, ammission diagnosis and procedures performed are summarized in Figure 5 and Figure 6.

### 3.4. Surgical Hospitalization Rates

**Figure 7:**
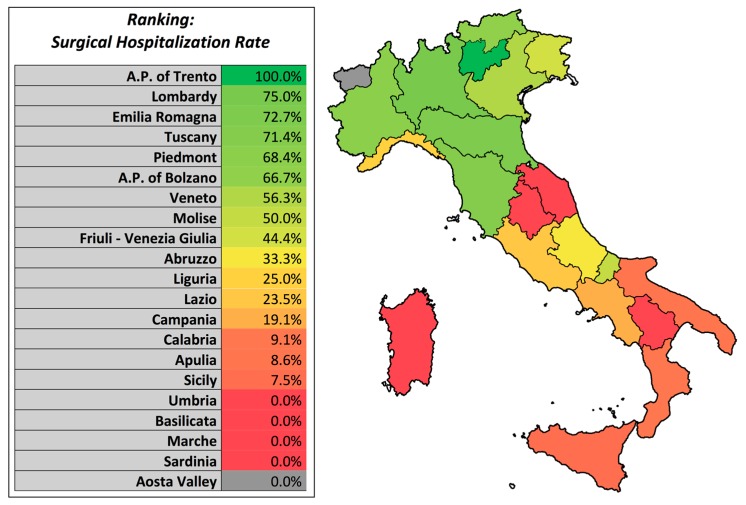
Surgical hospitalization rates ranking—a geographical representation. The colour gradient from dark green to light green, to yellow and finally to red and dark red represents the percentage of surgical hospitalizations for patients aged 0–14 years in the Italian regions due to shoulder dislocation, with dark green being the regions with the highest percentage of surgical hospitalizations (A.P. Trento) and dark red the regions with the lowest percentage of surgical hospitalizations (Sardinia, Marce, Basilicata, Umbria). The grey area (Aosta Valley) represents the only region with no hospitalizations for shoulder dislocation in the study period.

Data about surgical/non-surgical hospitalization rates showed that only one third (36.3%) of the hospitalizations required a surgical treatment. A ranking of the surgical hospitalization rates by region in summarized in Figure 7.

225 patients (65.4%) underwent hospitalization for a non-surgical treatment, which represented an incidence of 0.19 every 100,000 Italian inhabitants of the same age class. 119 patients (34.6%) underwent hospitalization for a surgical treatment, which represented an incidence of 0.11 surgery for shoulder instability every 100,000 Italian inhabitants of the same age class. In the surgical group, 47 patients underwent shoulder arthroscopy over the 14-year study period.

### 3.5. Public or Private Hospitalization

From 2001 to 2014, the average incidence rate of private hospitalization to treat shoulder instability in paediatric patients was 2.6% (9 out of 344).

### 3.6. Length of the hospitalization

The median length of hospitalisation was 1.2 days (range, 1–2.4 days). The median length of hospitalisation annually is described in Table 3.

## 4. Discussion

This registry study shows that the mean rate of hospitalizations in Italy for shoulder dislocation in patients under 14 years of age was 0.3 for every 100,000 inhabitants of the same class of age. The most common treatment was a closed reduction (60.8%), followed by open stabilization (16.6%) and arthroscopic procedures (13.7%) (Figure 4).

The majority of subjects received hospitalization in their own region of domicile.

Emilia-Romagna made the greatest number of extra-regional operations (13 patients). Aosta Valley was the only region to not perform any hospitalization for shoulder dislocation.

Different authors have found that the risk to re-dislocate is inversely linked to age at the period of the first dislocation, with the risk being highest for patients just under the age of 18 [21,25,26,44,45,46,47,48,49]. Because of the high recurrence rates for dislocations in young patients and the risk with each dislocation of soft or bony lesions, some authors prefer initial surgical stabilization [44].

Good outcomes after conservative treatment were reported for many orthopaedic conditions affecting children and young adolescents, including first-time dislocation of patellofemoral joints [50]. Considering shoulder dislocation, good outcomes for conservative procedures in paediatric subjects have been reported, with recurrence rates ranging between 6% and 33% [21,51,52]. Evaluating three patients younger than 14 years at the period of the first dislocation, Postacchini et al. [21] found that only one re-dislocated. A Bankart lesion was found in this patient. In a slightly bigger series of cases (14 patients), Cordischi et al. showed that re-dislocation occurred in 21% of skeletally immature patients with a primary conservative treatment. Surprisingly, no Bankart lesion was found in these patients. The authors have suggested that primary traumatic shoulder dislocation in subjects with immature skeletons should be considered a distinct pathoanatomical entity, as the greater elasticity of the articular capsule and the glenoid labrum of these patients are less inclined to experience injuries after trauma. Furthermore, in skeletally immature patients, the articular capsule inserts really near to the border of the glenoid and might have a greater tension, preventing redislocations [21,53,54]. Faster recovery and better functional outcomes have also been reported for skeletally immature subjects treated conservatively, compared to patients undergoing surgery [51,55]. On the other hand, two studies described a 100% incidence of redislocations for conservative procedures of shoulder dislocation in skeletally immature subjects [22,56].

The present registry study shows that in Italy between 2001 and 2014, shoulder dislocations in paediatric patients were most commonly treated conservatively (60.8% of the hospitalized patients).

We are not aware of any available national database or registry on hospitalization for shoulder dislocation in paediatric subjects to compare data.

Over the study period, more than 75% (*n* = 264) of hospitalizations to treat shoulder instability were performed in males from 12 to 14 years of age. This is the age in which children increase their participation in sports activities and this could be the reason for increased shoulder dislocation in this category of patients.

Regions from the centre and the southern part of Italy performed the majority of the hospitalizations for closed shoulder reduction. Often, these hospitalizations can be avoided as they don’t influence outcome and are much more expensive than outpatient care. Notably, the number of hospitalizations for closed shoulder reduction is decreasing all over the country. On the other side, hospitalization should be warranted for patients undergoing surgical treatment. In the northern part of Italy, the majority of the hospitalizations were performed for surgical treatment.

Our research has some limits. Firstly, it is based on administrative data from different regions and hospitals. The International Classification of Diseases 9 (ICD-9)was used for all the reported cases. Unfortunately, the ICD-9 did not allow us to differentiate between unidirectional and multidirectional shoulder dislocation. As so many different hospitals were involved, it is practically impossible to spot mistakes in diagnoses or coding. Furthermore, no data was provided for the type of surgical stabilization procedure (e.g., soft tissue vs bony procedures) of concomitant injuries.

## 5. Conclusions

In conclusion, our registry research shows that the mean rate of hospitalizations in Italy for shoulder dislocation in patients with less than 14 years was 0.3 for every 100,000 inhabitants of the same class of age. The most common treatment was a closed reduction (60.8%), followed by open stabilization (16.6%), and arthroscopic procedures (13.7%). Regions from the south and the centre are marked by a higher rate of hospitalization for closed shoulder reduction, which should be replaced, if possible, with outpatient care.

## Figures and Tables

**Table 1:** Number of hospitalizations, female vs male.

N° of HOSPITALIZATIONS PER YEAR—NATIONAL POPULATION																
	2001	2002	2003	2004	2005	2006	2007	2008	2009	2010	2011	2012	2013	2014	Total (ABS)	Total (%)
**Male**	24	21	16	18	18	25	16	17	18	9	15	12	10	12	231	67.15%
**Female**	8	10	6	9	9	9	7	13	8	6	5	5	9	9	113	32.85%
***Total***	32	31	22	27	27	34	23	30	26	15	20	17	19	21	344	100.00%
															**Avg**	
**Male/Female Ratio**	3	2.1	2.67	2	2	2.78	2.29	1.31	2.25	1.5	3	2.4	1.11	1.33	**2.12**	

**Table 2:** Number of procedures per 100,000 inhabitants.

N° OF PROCEDURES PER 100.000 INHABITANTS (0–15 YEARS OLD)															
	2001	2002	2003	2004	2005	2006	2007	2008	2009	2010	2011	2012	2013	2014	Avg
**0–15 years old Population (ISTAT)**	8,109,389	8,148,138	8,190,349	8,255,712	8,283,936	8,321,900	8,367,043	8,428,708	8,477,937	8,513,222	8,325,217	8,348,338	8,448,133	8,383,122	
**N° Hospitalizations per 100.000 inh (0–15 y old)**	0.39	0.38	0.27	0.33	0.33	0.41	0.27	0.36	0.31	0.18	0.24	0.2	0.22	0.25	0.3

**Table 3:** Hospitalization length.

HOSPITALIZATION LENGTH (Days)—NATIONAL POPULATION															
	2001	2002	2003	2004	2005	2006	2007	2008	2009	2010	2011	2012	2013	2014	Avg
**Hospitalization Lenght (days**)	2.4	1.1	1.2	1	1.1	1.2	1.1	1	1	1.1	1.1	1.1	1.1	1	1.2

**Table 4:** Number of hospitalizations per year, macro-area.

N° of HOSPITALIZATIONS PER YEAR—MACROAREA (North-Center-South)																
	2001	2002	2003	2004	2005	2006	2007	2008	2009	2010	2011	2012	2013	2014	Total (ABS)	Total (%)
**North**	5	10	5	5	9	12	8	12	14	6	8	12	11	12	129	37.50%
**Centre**	2	4	2	4	6	9	2	2	1	3	5	1	2	3	46	13.40%
**South**	25	17	15	18	12	13	13	16	11	6	7	4	6	6	169	49.10%
***Total National***	32	31	22	27	27	34	23	30	26	15	20	17	19	21	344	100.00%

**Table 5:** Data on interregional migration, regional, and extra-regional hospitalizations.

*Migration*	Piedmont	Aosta Valley	Lombardy	A.P. of Bolzano	A.P. of Trento	Veneto	Friuli - Venezia Giulia	Liguria	Emilia Romagna	Tuscany	Umbria	Marche	Lazio	Abruzzo	Molise	Campania	Apulia	Basilicata	Calabria	Sicily	Sardinia	Total Hospitalization performed	Extra-Regional Hospitalization (ABS)	Extra-Regional Hospitalization (%)	Regional Hospitalization (ABS)	Regional Hospitalization (%)
**Piedmont**	18	0	2	0	0	0	0	1	1	1	0	0	0	0	0	0	0	0	0	0	0	23	5	21.74%	18	78.26%
**Aosta Valley**	1	0	0	0	0	0	0	0	0	0	0	0	0	0	0	0	0	0	0	0	0	1	1	100.00%	0	0.00%
**Lombardy**	0	0	32	0	0	0	0	0	0	0	0	0	0	0	0	1	0	0	0	0	0	33	1	3.03%	32	96.97%
**A.P. of Bolzano**	0	0	0	6	0	0	0	0	0	0	0	0	0	0	0	0	0	0	0	0	0	6	0	0.00%	6	100.00%
**A.P. of Trento**	0	0	0	0	1	3	0	0	0	0	0	0	0	0	0	0	0	0	0	0	0	4	3	75.00%	1	25.00%
**Veneto**	0	0	1	2	0	13	2	0	1	0	0	0	0	0	0	0	0	0	1	0	0	20	7	35.00%	13	65.00%
**Friuli - Venezia Giulia**	0	0	0	0	0	0	4	0	0	0	0	0	0	0	0	0	0	0	0	0	0	4	0	0.00%	4	100.00%
**Liguria**	0	0	0	0	0	0	0	4	0	4	0	0	0	0	0	0	0	0	0	0	1	9	5	55.56%	4	44.44%
**Emilia Romagna**	0	0	3	0	0	0	0	0	11	0	0	0	0	0	0	0	0	0	0	0	0	14	3	21.43%	11	78.57%
**Tuscany**	1	0	0	0	0	0	0	0	1	13	0	0	0	0	0	0	0	0	0	0	0	15	2	13.33%	13	86.67%
**Umbria**	0	0	0	0	0	0	1	0	3	1	1	0	0	0	0	0	0	0	0	0	0	6	5	83.33%	1	16.67%
**Marche**	0	0	0	0	0	0	0	0	1	0	0	3	0	0	0	0	0	0	0	0	0	4	1	25.00%	3	75.00%
**Lazio**	0	0	2	0	0	0	1	0	1	1	0	0	18	1	0	0	1	0	0	0	0	25	7	28.00%	18	72.00%
**Abruzzo**	0	0	0	0	0	0	0	0	0	0	0	0	0	3	1	0	0	0	0	0	0	4	1	25.00%	3	75.00%
**Molise**	0	0	0	0	0	0	0	0	1	0	0	0	0	0	1	0	0	0	0	1	0	3	2	66.67%	1	33.33%
**Campania**	0	0	0	0	0	0	0	0	1	0	0	0	0	1	0	46	1	1	0	0	0	50	4	8.00%	46	92.00%
**Apulia**	0	0	1	0	0	1	0	0	1	0	0	0	1	1	0	0	35	0	0	0	0	40	5	12.50%	35	87.50%
**Basilicata**	0	0	0	0	0	0	0	0	0	0	0	0	0	0	0	0	0	2	0	0	0	2	0	0.00%	2	100.00%
**Calabria**	0	0	0	0	0	0	0	0	1	0	0	0	0	0	0	0	0	0	10	0	0	11	1	9.09%	10	90.91%
**Sicily**	0	0	1	0	0	0	0	0	1	1	0	0	1	0	0	0	0	0	0	49	0	53	4	7.55%	49	92.45%
**Sardinia**	0	0	1	0	0	0	0	0	0	0	0	0	0	0	0	0	0	0	0	0	9	10	1	10.00%	9	90.00%
